# Estimation of Daily Terrestrial Latent Heat Flux with High Spatial Resolution from MODIS and Chinese GF-1 Data

**DOI:** 10.3390/s20102811

**Published:** 2020-05-15

**Authors:** Xiangyi Bei, Yunjun Yao, Lilin Zhang, Yi Lin, Shaomin Liu, Kun Jia, Xiaotong Zhang, Ke Shang, Junming Yang, Xiaowei Chen, Xiaozheng Guo

**Affiliations:** 1 State Key Laboratory of Remote Sensing Science, Faculty of Geographical Science, Beijing Normal University, Beijing 100875, China; xiangyibei@mail.bnu.edu.cn (X.B.); jiakun@bnu.edu.cn (K.J.); xtngzhang@bnu.edu.cn (X.Z.); shangke@mail.bnu.edu.cn (K.S.); julming@mail.bnu.edu.cn (J.Y.); chen_xiaowei@mail.bnu.edu.cn (X.C.); boyxiaozheng@mail.bnu.edu.cn (X.G.); 2 Faculty of Geo-Information and Earth Observation (ITC), University of Twente, 7500 AE Enschede, The Netherlands; l.zhang-2@utwente.nl; 3 School of Earth and Space Sciences, Peking University, Beijing 100871, China; yi.lin@pku.edu.cn; 4 State Key Laboratory of Earth Surface Processes and Resource Ecology, Faculty of Geographical Science, Beijing Normal University, Beijing 100875, China; smliu@bnu.edu.cn

**Keywords:** terrestrial latent heat flux, data fusion, high spatiotemporal resolution, MODIS, Chinese GF-1 WFV

## Abstract

Reliable estimates of terrestrial latent heat flux (LE) at high spatial and temporal resolutions are of vital importance for energy balance and water resource management. However, currently available LE products derived from satellite data generally have high revisit frequency or fine spatial resolution. In this study, we explored the feasibility of the high spatiotemporal resolution LE fusion framework to take advantage of the Moderate Resolution Imaging Spectroradiometer (MODIS) and Chinese GaoFen-1 Wide Field View (GF-1 WFV) data. In particular, three-fold fusion schemes based on Enhanced Spatial and Temporal Adaptive Reflectance Fusion Model (ESTARFM) were employed, including fusion of surface reflectance (Scheme 1), vegetation indices (Scheme 2) and high order LE products (Scheme 3). Our results showed that the fusion of vegetation indices and further computing LE (Scheme 2) achieved better accuracy and captured more detailed information of terrestrial LE, where the determination coefficient (R^2^) varies from 0.86 to 0.98, the root-mean-square error (RMSE) ranges from 1.25 to 9.77 W/m^2^ and the relative RSME (rRMSE) varies from 2% to 23%. The time series of merged LE in 2017 using the optimal Scheme 2 also showed a relatively good agreement with eddy covariance (EC) measurements and MODIS LE products. The fusion approach provides spatiotemporal continuous LE estimates and also reduces the uncertainties in LE estimation, with an increment in R^2^ by 0.06 and a decrease in RMSE by 23.4% on average. The proposed high spatiotemporal resolution LE estimation framework using multi-source data showed great promise in monitoring LE variation at field scale, and may have value in planning irrigation schemes and providing water management decisions over agroecosystems.

## 1. Introduction

Terrestrial latent heat flux (LE), which refers to the heat flux transferred from the land surface to the atmosphere through soil evaporation, vegetation transpiration and interception, is an essential component for characterizing the global and regional hydrological budget, energy redistribution and carbon cycles [1,2]. The accurate estimation of terrestrial LE at high spatial and temporal resolution is of great significance for a wide range of applications [3]. For instance, robust and reliable acquisition of LE is an important prerequisite for water and soil conservation assessment, water resource management and terrestrial ecosystem monitoring at the field scale [4,5]. Currently, ground-based measurements such as the eddy covariance (EC) method can provide continuous observations of LE [6,7]. However, sparse point measurements prohibit the adequate depiction of LE variations, given the mismatch between small point and large spatial scales [8].

Remote sensing has long been regarded as the most feasible and efficient method to provide detailed and timely information on ecosystem dynamics, which can be used to estimate terrestrial LE [9,10]. Many moderate-resolution sensors (~1 km), such as the Moderate Resolution Imaging Spectroradiometer (MODIS) and Advanced Very High-Resolution Radiometer (AVHRR), can cover a global surface on a daily revisit and have gained popularity worldwide with public access, but their coarse resolutions strongly limit their applications to meet the accurate LE mapping at field or local scales. In contrast, the remotely sensed images acquired from Landsat series satellites Operational Land Imager (OLI) and Indian Remote Sensing Satellite (IRS) provide high spatial resolution ranging from 6 m to 30 m, which contributes more to LE change detection. Nevertheless, their lengthy revisit intervals (e.g., Landsat/OLI: 16 days, IRS:26 days) extremely constrain their utility in monitoring the rapid change of LE. Despite new remote sensing systems, such as Sentinel-2, improving spatial or temporal coverage over earlier satellite products, the acquiring of satellite images with both high frequency and high spatial resolution is still challenging. [11,12]. Therefore, there is an unprecedented opportunity for combining the advantages of different data sources to achieve both high temporal and spatial resolution images [13].

During the last few decades, several satellite-based data fusion approaches have been developed to bridge the gap between the high and low spatial resolution data and improve the spatiotemporal consistencies by fusion multi-scale products [14,15]. These general approaches have been successfully carried out to monitor terrestrial LE variances. For example, Xu et al. [16] presented a Multi-Resolution Tree (MRT) method to merge MODIS- and Landsat-based LE products and significantly improved the consistency and decreased the uncertainties of individual satellite products. In another approach, Bhattarai et al. [17] proposed a simple linear regression-based fusion model to integrate the seasonal ET at Landsat-like scale and enhance the availability of cloud-free Landsat images. However, these data fusion approaches are not suitable for providing continuously spatial and temporal coverage and reconstructing the high spatiotemporal terrestrial LE dynamics. 

Recently, one of the most widely used data fusion algorithms is the Spatial and Temporal Adaptive Reflectance Fusion Model (STARFM) proposed by Gao et al. [18], aimed to enhance both spatial and temporal resolution to produce daily reflectance data simultaneously from the one MODIS and Landsat imagery. Since then a number of spatiotemporal fusion methods have been proposed [19,20]. For example, the enhanced STRFM (ESTARFM) is developed by Zhu et al. [21] to reduce the uncertainties in complicated heterogeneous. Hilker et al. [22] proposed Spatial Temporal Adaptive Algorithm for mapping Reflectance Change (STAARCH) to handle the transient disturbance on the original model. An Improved Flexible Spatio-temporal Data Fusion method (IFSDAF) is devised by Liu et al. [23] for producing the Normalized Difference Vegetation Index (NDVI) time series with high spatial and temporal resolution. Moreover, Zhao et al. [24] presents a Robust Adaptive Spatial and Temporal Fusion Model (RASTFM) to address complex land surface change. Among these spatiotemporal fusion methods, ESTARFM has been successfully applied to estimate daily LE at field scale, and shows satisfactory accuracy especially in the highly heterogeneity spatial area [25]. The most significant improvement of ESTARFM is to introduce a conversion coefficient to deal with the spatial information accurately and efficiently [26]. Consequently, ESTARFM is generally recommended and chosen to reconstruct daily LE maps.

From the current spatiotemporal data fusion research, the following issues can be concluded: Firstly, despite the rapid development of newly launched satellite sensors, the input datasets only cover the MODIS and Landsat imagery. Alternatively, the Chinese GaoFen-1 Wide Field View (GF-1 WFV) sensor provided highly valuable data with the fine resolution (~16 m/4 days) for monitoring the terrestrial spatial-temporal variations of LE [27]. Thus, there is an urgent need for exploring the potential of emerging satellite sensors to facilitate LE estimation at high spatial resolution. Secondly, all these approaches have been mainly used to primarily predict the high spatial-temporal surface reflectance and then utilize these outputs to calculate biophysical indices, where LE product have rarely been involved in the fusion process as one objective. Herein, GF-1 and MODIS data were used to generate the high spatial-temporal resolution LE products in three-fold schemes: (1) fusion of GF-1 and MODIS surface spectral reflectance data to derive the Vegetation Index (VI), and then use these parameters to generate LE product. (2) fusion of VI derived by GF-1 and MODIS surface spectral reflectance data, and then generate LE product. (3) directly fusing the GF-1 and MODIS LE products. 

In this paper, we used the ESTARFM model to fuse the GF-1 and MODIS data and generate the high spatial temporal resolution LE product at threefold schemes. We had three major objectives. First, we evaluated the satellite-derived LE products against the flux tower sites. Second, we compared three fusion schemes and then the optimal scheme was chosen to generate time-series LE product at 16 m resolution in 2017. Third, we assessed the predicted LE product with coarse scale LE imagery and EC observations in flux tower sites.

## 2. Materials and Methods

### 2.1. Study Area and Ground-Based Observations

**Figure 1:**
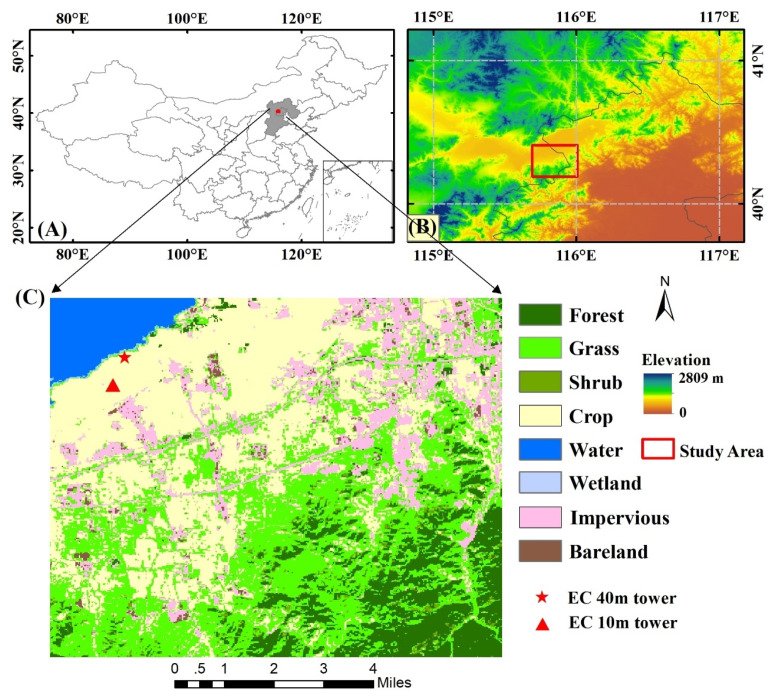
Maps showing (**A**) the location of the study area (**B**) the digital elevation model (DEM) data of the study site (**C**) the distribution of land cover type and the location of EC towers.

A case study was selected to evaluate the effectiveness of the proposed three LE fusion schemes for the GF-1 WFV and MODIS data. The study area is located in the Hai River Basin of Northern China (Figure 1), characterized by the continental semi-humid and semi-arid climate. The average mean temperature was 10.4 °C and the annual mean precipitation was approximately 500 mm, which commonly occurred during the summer season from June to August. The land cover types consist of cropland, forest, grassland, shrubland, wetland, water, impervious surface and bare land developed by the FROM-GLC (Finer Resolution Observation and Monitoring of Global Land Cover) product with 30-m resolution [28]. Therefore, the complex land cover compositions and heterogeneity underneath the surface are extremely appropriate for the application of LE estimation.

The study area contains two flux tower situated at the cropland with an irrigated corn area nearby, namely Huailai station - EC system - 10 m tower at Site 1 (115.788E, 40.3491N; hereafter EC 10 m site) and Huailai station - EC system - 40 m tower at Site 2 (115.7923E, 40.3574N; hereafter EC 40 m site), which were obtained from the multi-scale surface flux and meteorological elements observation dataset in the Hai River Basin [29]. Daily LE data (W/m^2^) was calculated from every half-hour ground-based LE flux measurements based on the EC system from 2014 to 2017. To estimate the flux footprint, we used a Eulerian analytic method proposed by Kormann et al. [30]. Specifically, the GF-1 LE maps were overlapped with the footprint weighted maps of EC. Each pixel of footprint weight within the source area was summed to acquire the validation pixels [31]. Because of the energy nonclosure limitation, the latent heat fluxes and water vapor may be underestimated, so we used the method proposed by Twine et al. [32] to correct the LE measurements at two sites:

(1) $$LE_{cor}=\frac{R_{n}-G_{s}}{LE_{obs}+H_{obs}}\times LE_{obs,}$$

where $LE_{cor}$ is the corrected latent heat flux, and $LE_{obs}$ and $H_{obs}$ are the observed latent heat flux and sensible heat flux based on the EC system, respectively.

The following meteorological data were acquired from the automatic weather station (AWS) including air temperature (Ta), wind speed (Ws), relative humidity (RH), soil heat flux (Gs), shortwave solar radiation (Rs). All meteorological data were measured at 10-min intervals and then aggregated into daily or 8-day mean values.

### 2.2. Satellite Data

The Chinese GF-1 is known as the first satellite of the China High Resolution Earth Observation System, launched at the Jiuquan Satellite Launch Centre of China in April 2013 [33]. The GF-1 carries six cameras, including two Panchromatic Multi-spectral (PMS) cameras with 2 m/8 m spatial resolution and four WFV cameras with 16 m spatial resolution. The WFV cameras have the four spectral channels: blue, green, red and near-infrared bands, and spectral range from 450 to 890 nm [34]. The specific information of the GF-1 product is listed in Table 1. In theory, GF-1 WFV has a 4-day revisit cycle by combining the four WFV cameras. Nevertheless, the unfavorable atmospheric conditions and cloud contamination contribute to occasional absence data for half a month. In this study, nineteen cloud-free GF-1 WFV images with four spectral bands were collected from January 2014 to November 2017. We obtained level 1 GF-1 WFV products, which have been processed by relative radiation correction. The systematic atmospheric correction and precision geometric correction was conducted on the ENVI platform. In addition, the Landsat 8 OLI product was selected as the reference data for the precision geometric correction to improve the positioning accuracy of GF-1 products.

MODIS spectral reflectance (SR) product (MOD09GA) provides MODIS band 1–7 surface reflectance at a 500-m daily resolution acquired from Land Processes Distributed Active Archive Center (LP DAAC). It has corrected the effects of atmospheric gases and aerosols and gridded these factors into the sinusoidal projection. In this study, the daily scenes of h26v04 during the period of 2014–2017 were collected, of which four spectral bands including bands 1 (red), 2 (near-infrared), 3 (blue) and 4 (green) were specifically selected and reordered similar to GF-1 WFV bands. Table 1 lists the bandwidths for GF-1 and MODIS. To match the GF-1 WFV data and MODIS SR data in the same projection, we used the MODIS Reprojection Tool (MRT) to convert the file format (HDF-EOS to GeoTIFF) and processed the projection to Universal Transverse Mercator (UTM) coordinate system. Then, the MODIS data was subset to the extent of the study area and resampled to the 16m resolution using the bilinear interpolation method to meet the model input requirement. In addition, the outlier caused by cloud shadow contaminated were removed. Since we highlight acquiring the spatial and temporal continuous LE products, the cloud-free image close to acquisition dates provide complementary information to gap-filling the miss pixels. 

The shortwave broadband albedo as one of the auxiliary satellite data was obtained from the Global LAnd Surface Satellite (GLASS) albedo product, which has been generated and released to the public in November 2012 [35]. GLASS albedo product with 5 km and 8-day resolution is produced by the AVHRR and MODIS data, which present the satisfactory spatial-temporal coverage and reasonable consistency with ground measurements. The cross-comparison also demonstrated that GLASS albedo product has higher accuracy and lower root mean square error (RMSE) than MODIS albedo product and better captured the albedo spatiotemporal pattern [36]. The digital elevation model (DEM) data at 250 m spatial resolution acquired from 90 m Shuttle Radar Topography Mission (SRTM) images (version 004), which was used to calculate surface Net Radiation (Rn) in our study.

### 2.3. Methods

#### 2.3.1. Implementation and Evaluation of the Three Fusion Schemes

**Figure 2:**
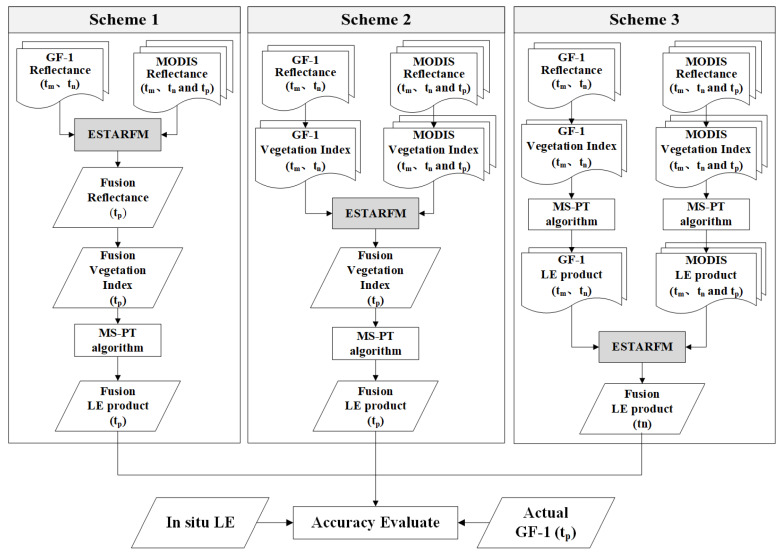
Flowchart of the proposed three fusion schemes using GF-1 and MODIS data.

A schematic overview of three fusion schemes is described below, Figure 2 shows the schematic flowchart of the proposed methodology. 

(1) Scheme 1: Given that a clear sky GF-1 WFV images and MODIS SR images are acquired on date tm and tn, ESTARFM simulates GF-like surface reflectance for date tp based on the two pairs GF-1/MODIS SR images on date tm and tn and another MODIS SR image on tp. Then, based on the simulated GF-like SR, the NDVI image on date tp was calculated. Finally, simulated VI combined with the meteorological parameters as inputs to generate the simulated 16m LE using the Modified-Satellite Priestley–Taylor (MS-PT) algorithm.

(2) Scheme 2: Instead of fusion SR products, first derive VI image based on two pairs GF-1/MODIS image on date tm and tn and MODIS image on date tp. Then, the simulated VI is used to calculate the 16m LE product. 

(3) Scheme 3: Directly generating GF-1/MODIS LE product. Then the GF-1/MODIS LE on date tm and tn and MODIS image on date tp are used to predict the 16m LE product based on ESTARFM.

To evaluate the three fusion schemes, LE obtained from the GF-1 data on the date tp was preserved as the reference LE for validation and was not used as input for fusion model (e.g., on the date in Year/DOY format, 2015/010; 2015/269; 2016/121; 2016/149; 2017/091; 2017/133; 2017/193); Then, we randomly selected 10% of the total pixels on a pixel-by-pixel basis to validate the simulated three LE products. The scatterplots were used to compare three fusion schemes and then the scheme with higher accuracy was chosen to generate time-series LE product in 2017. The performance of LE product after fusion is evaluated using ground-measured flux data.

#### 2.3.2. ESTARFM

The Enhanced Spatiotemporal Fusion model (ESTARFM) can capture spatial changes of reflectance from two pairs GF-1/MODIS images and another MODIS image designed by [21]. Compared to the original STARFM model [37], the ESTARFM introduced the conversion coefficient to calculate the ratio of changes between the fine (GF-1) and coarse (MODIS) resolution pixels and improved the accuracy of simulated product over the heterogeneous landscapes [26]. Although ESTARFM were originally developed to generate the surface reflectance data from Landsat and MODIS images, this algorithm can be also expanded to MODIS and GF-1 fusion applications given the similar bandwidths. The detailed information of the ESTARFM algorithm is provided in Appendix A.

#### 2.3.3. MS-PT Algorithm

We produced the GF-1 and MODIS LE product using the Modified-Satellite Priestley-Taylor (MS-PT) algorithm proposed by Yao et al. [38] It has been proved that the MS-PT algorithm decreases the root-mean-square error (RMSE) approximately 5 W/m^2^ in daily LE estimation compared to the Priestley–Taylor-based (PT–JPL) algorithm [9]. Previous studies also demonstrate that the MS-PT algorithm has a good performance and provides reliable LE estimations over multiple biomes [39]. This algorithm introduced the apparent thermal inertia (ATI) as the primary parameters for characterizing the soil moisture constraints to minimize the computational complexities of aerodynamic and surface resistance. The input parameters require only the air temperature (Ta), the ATI derived by the diurnal air temperature range (DT), the net radiation (Rn) and the satellite VI (NDVI). We obtained Ta and DT from the AWS site and calculated Rn based on the method of Wang et al. [40] Appendix B. provides detailed descriptions of the MS-PT algorithm.

#### 2.3.4. Accuracy Assessment Method

The following statistical criteria were used to assess the accuracy of fusion approach. The coefficient of determination (R^2^) describes that the observation variation can be explained by the predicted model, the root-mean-square error (RMSE) and the relative RSME in percentage (rRMSE) measure the deviation between the observed value and the predicted value, and the bias reflects the mean difference between the observed value and predicted value. 

## 3. Results

### 3.1. Validation of Satellite-Derived Latent Heat Products at the Flux Tower Sites

**Figure 3:**
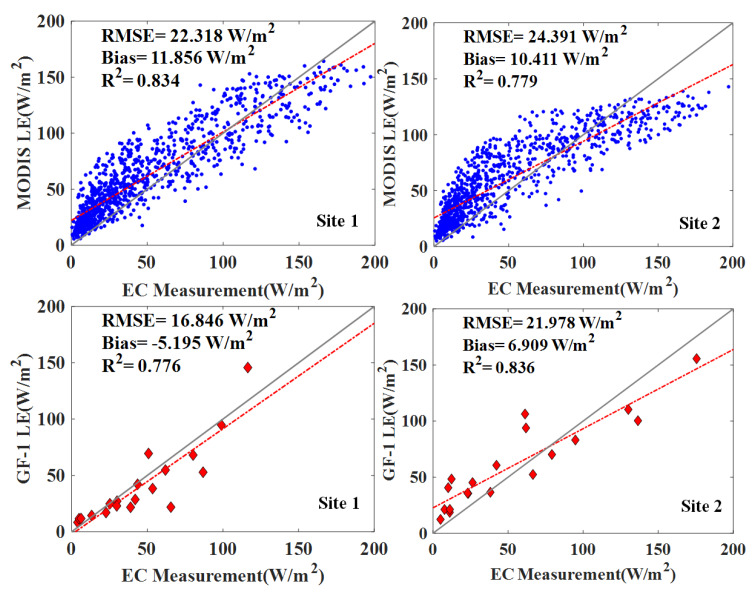
Scatterplots of the satellite derived LE products and ground measurements at daily scale during 2014–2017. The blue circle and red diamond represent the MODIS and GF-1-derived LE products, respectively.

Figure 3 shows the scatterplots of the comparison between the estimated daily LE and the corresponding ground observations. The blue circle and red diamond represent the MODIS and GF-1-derived LE products, respectively. At the site scale, two different LE products illustrate substantial differences. The bias in the deviation of MODIS LE product from the EC measurements varies from 10.4 to 11.9 W/m^2^, the RMSE varies from 22.3 to 24.4 W/m^2^, the MS-PT algorithm explains approximately 80% of LE variability given that R^2^ varies from 0.78 to 0.83. The GF-1 LE product shows generally favorable agreement with local measurements, with a lower bias of 7.1 and 7.8 W/m^2^ and RMSE of 18.0 and 21.6 W/m^2^. 

**Figure 4:**
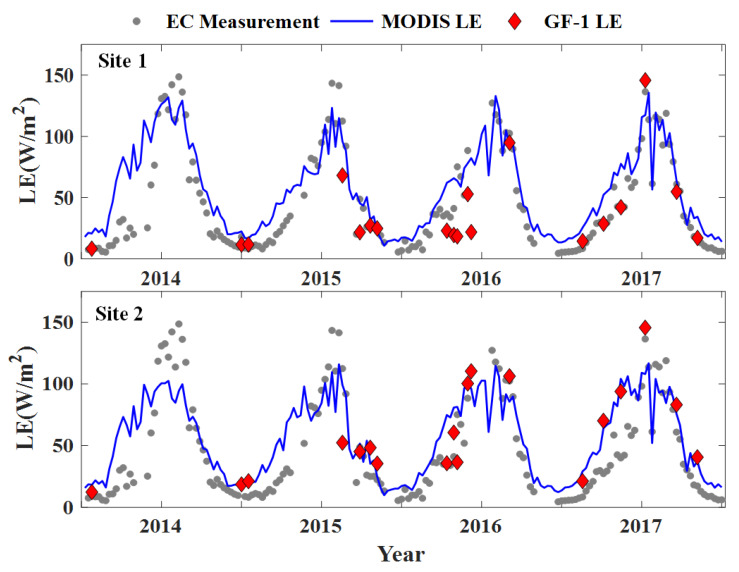
Intercomparison of 8-day averaged estimations and observations on time series of 2014–2017 for Site 1 (top panel) and Site 2 (bottom panel).

The 8-day averaged value of the simulations and observations using time series are compared in Figure 4. The interannual variation of LE presents a unimodal curve, reaching its peak value in summer and then decreasing in winter. In terms of the MODIS LE product, most values overestimated LE in the non-growing season and underestimated LE in the growing season by approximately 10–20 W/m^2^. In contrast, the GF-1 LE product exhibits better correlations with in situ observations. Unfortunately, only nineteen images are available owing to the frequent cloud contamination. Overall, the general correspondence supports the reliability of the MS-PT algorithm for monitoring land surface LE variation.

### 3.2. Comparisons of the Three Fusion Schemes 

**Figure 5:**
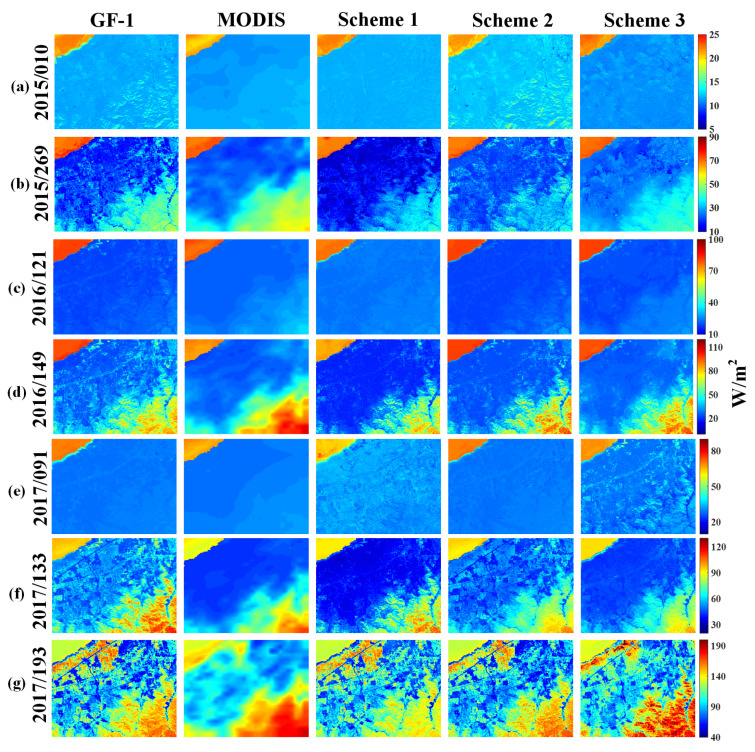
Spatial comparison of three fusion schemes estimated LE with the corresponding GF-1 and MODIS LE on the GF-1 available validation dates. The column order from the left to right: **(1)** actual reference GF-1 LE, **(2)** MODIS LE, **(3)** estimated LE for Scheme 1, **(4)** estimated LE for Scheme 2, **(5)** estimated LE for Scheme 3. The row order from the top to bottom: (**a**) 2015/010, (**b**) 2015/269, (**c**) 2016/121, (**d**) 2016/149, (**e**) 2017/091, (**f**) 2017/133, (**g**) 2017/193 (in Year/DOY format).

**Figure 6:**
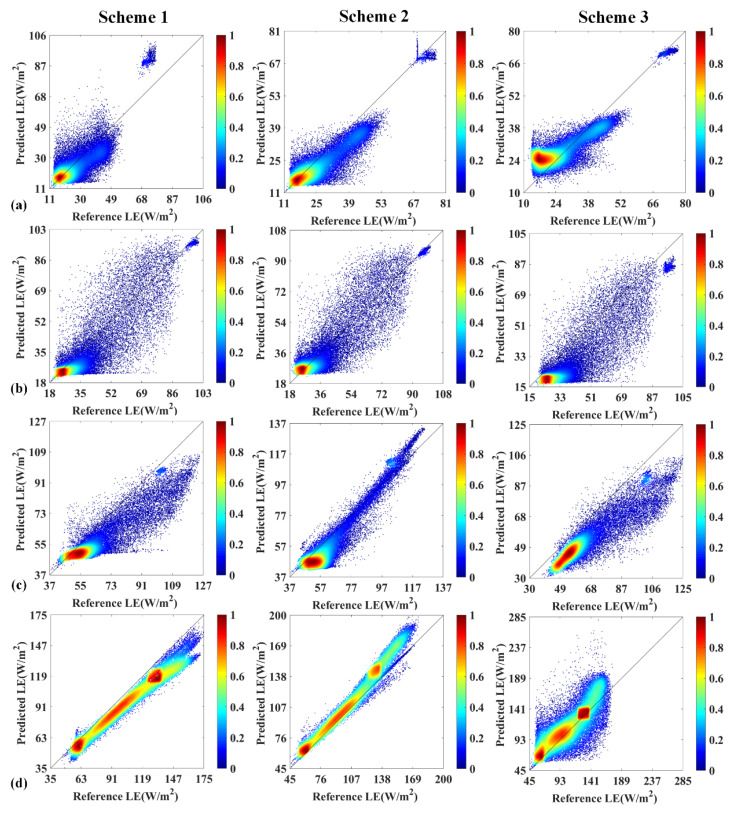
Scatter plots of comparison between the estimated LE vs. GF-1 reference LE on a pixel basis. (**a**) 2015/269, (**b**)2016/149, (**c**)2017/133, (**d**)2017/193 (in Year/DOY format).

Figure 5 shows the comparison of LE products estimated from the actual GF-1, MODIS data and three fusion schemes. Despite some differences, the predicted LE images successfully provided the finer spatial patterns and contained more detailed information than the corresponding MODIS images in different land cover types. One notices that the estimated LE from three schemes is similar to the actual GF-1 image and captured clear road, agricultural land and sparse vegetation patches. More importantly, the irrigation practices of cropland with relative high evapotranspiration are detectable in 2017/193, which can hardly be captured by MODIS imagery. The comparisons also show that Scheme 2 of simulated LE exhibits a better agreement with the actual GF-1 images, while the Scheme 3 with directly fused LE seems somewhat blurry with a less clear boundary, and presents poor performance and obvious differences. Because LE is relatively uniformly distributed over the area in the early season (2015/010, 2016/121, 2017/091), pixel by pixel comparison between three schemes and GF-1-based reference LE in the growing season are shown in Figure 6. The validation also indicates that the Scheme 2 points are closer to the 1:1 line than other schemes. 

**Figure 7:**
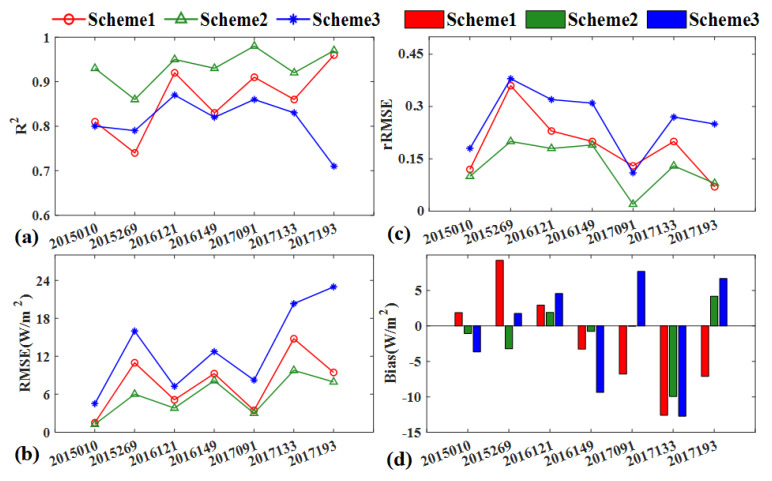
(**a**) R^2^, (**b**) RMSE, (**c**) rRMSE, (**d**) bias comparison between the estimated LE and the reference LE on the validation dates.

The comparison on the pixel basis between the different schemes predicted LE and the corresponding reference LE are provided in Figure 7. The relative RSME in percentage (rRMSE) is acceptable ranging from 12% to 36% for Scheme 1, 2% to 23% for Scheme 2 and 11% to 38% for Scheme 3, since the predicted LE of the validation dates is reconstructed by fusion model rather than directly acquired from the actual GF-1 imagery. Further examination of the consistency demonstrated that Scheme 2 of predicted LE based on the fusion of VI was superior to the Scheme 1 and 3, with relative higher R^2^ and lower RMSE and bias. The findings are in line with the emerging evidence that the Scheme 2-first index then blend produced higher accuracy than the Scheme 1-first blend then index, because of the less error propagation reported by Jarihani et al. [41]. The predicted error of Scheme 1 is slightly larger, which can be partially attributed to the effects of actual differences the sensor when ESTARFM is expanded to the MODIS and GF-1 imagery fusion applications. As NDVI and LE are highly correlated, it is reasonable that the performance of simulated LE strongly dependent upon the accuracy of predicted VI.

Specifically, the performance of Scheme 3 with directly fused LE leaves much to be desired, with the lowest R^2^ and highest RMSE, rRMSE and bias. On all validation dates, Scheme 3 consistently generated slighter poor results, with R^2^ of 0.61 to 0.87, RMSE of 4.5 to 22.97W/m^2^ and rRMSE of 0.11 to 0.38, compared to the reference LE. Especially on the growing season of 2015/259 and 2017/193 with the highly heterogeneous surface, Scheme 3 cannot capture the sharped LE changes and generally underestimated and had the larger errors than another two approaches. The result is reasonable because LE on the date of 2015/269 was predicted by the two pairs of GF-1 and MODIS LE imagery on the dates of 2015/227 and 2015/285. It is evident that terrestrial LE has great temporal changes during the long period time. The algorithm cannot accurately predict the short time changes and will thus blur the boundary of phenology and land cover change. Consequently, Scheme 2 has a higher accuracy to produce a satisfactory estimation of LE variation and then Scheme 2 was selected to generate the time series LE in 2017.

**Figure 8:**
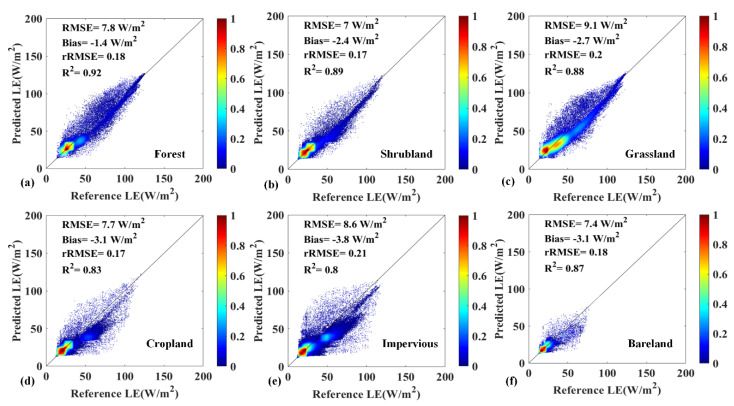
Scatter plots of comparison between estimated LE vs. GF-1 reference LE from Scheme 2 over different land cover types:(**a**) forest; (**b**) shrubland; (**c**) grassland; (**d**) cropland; (**e**) impervious; (**f**) bareland.

The scatter plots of different land cover types comparison based on the optimal Scheme 2 are further discussed in Figure 8. In general, most pixels followed the 1:1 line and have a good agreement. whereas most of the dispersions occur in impervious land (R^2^ = 0.8). The largest rRMSE (0.21) is likely due to the large spatial heterogeneity of mixed pixels of impervious area, which consists of roads, residences and cropland. The accuracy of fusion in forest, shrubland and grassland was relatively good (0.92 at forest, 0.89 at shrubland and 0.88 at grassland). 

### 3.3. Agreement of Fused LE with MODIS LE

**Figure 9:**
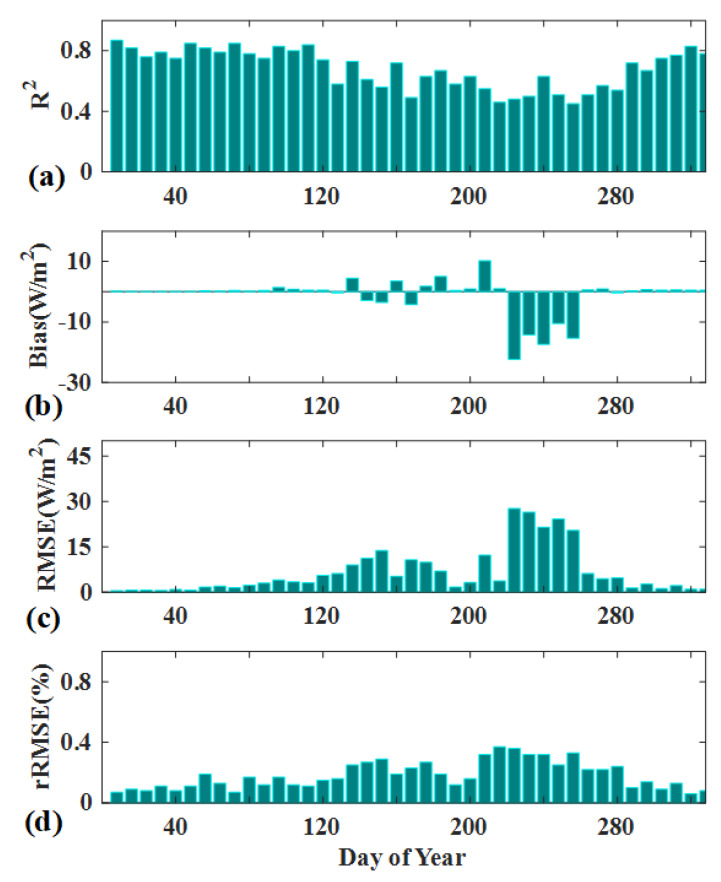
Comparisons of fused LE against MODIS LE when aggregate to MODIS gird. (**a**) R^2^, (**b**) bias, (**c**) RMSE, (**d**) rRMSE.

Given the fact that the six images of actual GF-1 data obtained from the 2017/041 to 2017/305 during the different span period, we further applied Scheme 2 to produce the daily LE in 2017. The GF-like LE products with 16-m resolution were aggregated to MODIS 500 m grids and the time series agreement with MODIS LE product was assessed in Figure 9. The results showed that synthetic LE presents a relatively good agreement with MODIS LE. R^2^ ranges from 0.48 to 0.88, RMSE was around 0.5–27.6 W/m^2^ and rRMSE are between 3% and 37%. However, there are still larger discrepancies during the growth season due to its high surface heterogeneity. The scale mismatch between the GF-like LE and MODIS LE, that might result in the large uncertainties, overestimate or underestimate of LE and even the systematic bias. Ke et al. [37] also reported the predicted LE based on Landsat-available data and MODIS LE data presents the fairly good consistency with average rRMSE around 22%. Although the comparison of estimated LE and MODIS LE could not reveal the capability in reconstructing the spatial detail, the utility of the fusion approach as one feasible and robust tool is clearly apparent.

### 3.4. Comparison of LE Products before and after Fusion

**Figure 10:**
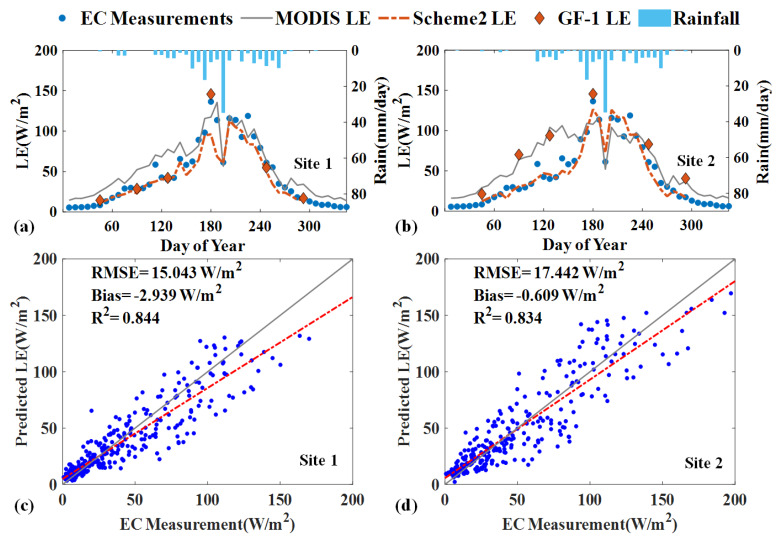
Comparisons between MODIS LE product, GF-1 LE Product and Scheme 2 simulated LE product with EC measurements. The top panel shows the time series of LE in 8-day averaged scale at (**a**) Site 1 (**b**) Site 2, the bottom panel shows the scatter plots of predicted LE with EC measurements in a daily scale at (**c**) Site 1 (**d**) Site 2.

The time series comparison of in situ data, the estimated LE, the original GF-1 and MODIS LE at 8-day average timescale for site 1 and site 2 are shown in Figure 10a,b. It can be seen that the estimated LE and observed LE have a general consistent temporal variation, especially in the growing season around in July follows a clear two-peak pattern. The 8-day accumulate rainfall occurs during the summer season both for two sites. The estimated LE was better able to capture the notable trough response to a rainfall event in the peak of the growing season, which can be explained by the rainy condition decreased shortwave radiation [42]. It is evident that MODIS LE and GF-1 LE products also play an essential role in estimating time series LE to adjust the predicted imagery value. 

We further evaluate the performance of the predicted daily LE using Scheme 2 versus the EC measurements in 2017. As shown in Figure 10c,d, the scatter plots show good agreement between the estimated LE and the EC measurements and most of the pixels followed a 1:1 relationship. The more improvement was that the RMSE of the estimated LE significantly decreased compared to the original satellite-based products in Table 2. The R^2^ increased from 0.78 to 0.84, the RMSE dropped by 23.4% on average, and the bias decreased by approximately 59.2% after fusion. However, it should be noted that the capacity of capturing small fluctuation is limited when the transient changes are not recorded in the GF-1 imagery. Overall, the estimated LE was able to characterize the variation of LE and showed apparently agreed with the observed values, which are rarely characterized by the GF-1 LE product. 

## 4. Discussion

### 4.1. Terrestrial LE Estimation Differences Using Three Fusion Schemes

In this study, we compared three spatiotemporal fusion schemes and estimated daily terrestrial LE with the high spatiotemporal resolution by fusing MODIS and GF-1 products. Although the filed-scale LE images via three fusion schemes successfully captured detailed spatial variation at GF-like resolution, there was a substantial difference in simulating terrestrial LE among these schemes. In our study, we estimated satellite-based LE products using the MS-PT algorithm, in which NDVI was supposed to be one important indicator for LE variation. Quantitative comparisons demonstrated that fusion of VI and then computing LE (Scheme 2) consistently yields better results than the other plans. Accordingly, Scheme 2 directly generated LE based on the GF-1/MODIS NDVI, which can eliminate some of the effects of the clouds and aerosols contamination and showed better performance. Previous researches supported our findings and highlighted that the strategy of Index-then-Blend is generally recommended to integrate the higher-order product [23,43]. The advanced filters in scheme 2 could eliminate the noise and outliers, whereas non-linear changes on the raw reflectance bands would amplify errors in Scheme 1 [44]. In addition, there are higher computational efficiency and less error propagation in Scheme 2 because of the fusion of a single spectral band. The findings are consistent with Tian, et al. [45], who also compared similar approaches (Scheme 1 and Scheme 2) based on the STARFM model to predict a time series NDVI images and concluded that Scheme 2 performed much better than Scheme 1.

In contrast, Scheme 3 did not consider the temporal change in the sub-pixel landscapes resulting in the underestimate or overestimate, which was the main cause of its poor performance. Directly fused high order LE product could not handle the abrupt variance over different land cover types and thus increased uncertainties. Our results are in agreement with those of earlier findings from Ma, et al. [46], who successfully applied the ESTARFM to produce daily LE maps by integrating the MODIS and Landsat data and concluded that the IPFA (input parameter fusion approach—Scheme 2) outperformed the ETFA (evapotranspiration fusion approach—Scheme 3). Although several investigations have proposed the different LE estimation framework, the performance of LE by fusing MODIS and GF-1 product was seldom comprehensively assessed. Consequently, three proposed fusion schemes all provided a detailed description for characterizing the turbulent heat flux, in which Scheme 2 achieved better performance followed by Scheme 1 and Scheme 3.

### 4.2. Uncertainties of the Fused LE Estimates

The time series estimated LE obtained from Scheme 2 not only provided the detailed spatial information of LE variances, but partially improved the accuracy and explained more than 83% of the LE variability according to the validation with EC observations. However, the fused LE estimation still contains large uncertainties, which can be attributed to the quality of the satellite products and input meteorological data [47], scaling effects caused by spatial mismatch [48,49], the errors of the EC ground measurements [50] and the algorithm’s inherent limitations [51].

First, the accuracy of the LE estimation is highly dependent upon the quality of the satellite products and input forcing data. Due to the wide scanning width of the GF-1 WFV (800km) product, the geometric and geolocation errors caused by serious distortion are approximately 1–10 pixels [52]. Even though the geometric correction based on the rigorously calibrated images Landsat-8 was implemented using the ground control points, the precision of the geometric positioning still exists potential uncertainties [53]. For the MODIS product, although the unreliable data has already been removed according to the MODIS quality flags, the biases introduced by gap filling cannot be ignored. Moreover, the satellite data pre-processing such as reprojection and interpolation may be the possible reason to reduce the accuracy of LE estimation. Since the individual GF-1 and MODIS LE products are estimated by meteorological variables from in situ observations, the meteorological data obtained from the AWS system have inherent uncertainties of 5%~15% that will increase the uncertainties of LE estimation [54]. 

Second, the scaling effect due to the spatial mismatch between the flux tower and the satellite products is another problem [55,56]. The measuring height is 10 m and 40 m at site 1 and site 2 respectively, which has a significant impact on the footprint size. The EC flux tower represents a small scale of hundred meters, whereas the GF-1 based LE estimation is merely 16m. Direct comparison of the measured LE with satellite-based images will lead to a large discrepancy. Moreover, the heterogeneous landscape, complicated canopy structure and the stochastic nature of turbulence also explain the large uncertainties of satellite derived LE product compared to the in situ measurements [57].

Third, much has been written about substantial errors in EC measurements result from the instrument bias, energy imbalance and data processing [58,59]. As investigated by Liu et al. [29], EC 40 m flux tower (Site 2) is located in an irrigated corn area near a reservoir. The difference in their source areas and the large spatial discrepancy are the primary reasons causing the difference in measurements over two sites. In addition, the complexity of atmospheric conditions and wind patterns also influence the enclosure of the EC system. Even though the EC measurements are relatively accurate for evaluating LE estimates, they still contain the uncertainty of approximately 5–20% that have not been solved reasonably [29]. According to Foken [8], in the lower boundary, a typical EC system only captures small eddies and the large eddies can hardly be measured, which may lead to the energy imbalance problem. Although we corrected the LE value based on the method designed by Twine et al. [32], considerable uncertainties still exist in the correction. Data processing including averaging, interpolation and threshold filtering could also lead to the uncertainties, which will affect the accuracy of the resultant LE products.

Last, potential uncertainties of LE estimation may be inherited from the algorithm itself. Although previous studies confirmed that PT-based algorithms perform satisfactory predictive capabilities and simplified the input parameters, the water and vegetation constrain have large differences over various biomes and conditions [60]. The estimated LE obviously showed that the MS-PT algorithm usually overestimated LE in winter and spring and underestimated in the growing season, which is in accordance with the findings by Hao et al. [61] This occurred because the MS-PT algorithm uses the apparent thermal inertia (ATI) to reflect the water stress, which could not characterizes the soil evaporation process well especially during the irrigation season [62]. Another limitation of the MS-PT algorithm was that calibrating the coefficients using LE observations at different land cover types has not been considered. Thus, the considerable biases would transfer to the LE estimation.

### 4.3. Prospects and Limitations of This Study

Recently, a number of spatiotemporal fusion technology have been developed to generate LE products with high spatiotemporal resolution. This paper for the first time presents a comprehensive comparison of three different schemes, and investigated the optimal method to estimate daily terrestrial LE products at high spatial resolution. Results demonstrated the feasibility and robustness of Scheme 2 applied in fusion of GF-1 and MODIS data and the estimated LE showed generally corresponded well with EC measurements and MODIS LE products. Although this fusion method was originally designed for the Landsat and MODIS reflectance data, it can also be extended to serve other high-level product applications. Additionally, the most significant superiority of the fusion framework is generating continuous imagery with detailed spatial information, which provides a reference dataset for the management of water recourse and irrigation of cropland at filed or local scale.

Although this study has achieved comparable terrestrial LE estimation from the GF-1 and MODIS data, it is still constrained by three known limitations. Firstly, it developed a linear equation to solve the fine imagery, which may introduce unrealistic records due to the nonlinear changes and noises contained in the different sensors. Thus, a nonlinear model or an unmixing-based method should be involved in the ESTARFM model to reduce the uncertainties [14]. Secondly, it failed to recover the pixels that occur the abrupt land cover changes or disturbances. Emelyanova et al. [63] applied the ESTARFM in two different landscapes and found that the sudden changes and transitions in land cover have a significant effect on the fusion accuracy. Finally, it is time consuming (about 15 min for one sample). It should be noted that searching similar pixels require a relatively long processing time, especially where the spatial and temporal variances are large between the base and predicted images.

## 5. Conclusions

Accurate estimation of the terrestrial LE at high spatial and temporal scales can be of significant value in monitoring surface water balance and energy exchange. As the current LE products derived from satellite data generally have fine spatial resolution or high temporal resolution, the ESTARFM data fusion approach has shown promise in time continuous and robust LE estimation for combining the satellite data over the different spatiotemporal resolutions. This study showcased the comparison of three fusion schemes to integrate GF-1 WFV and MODIS data with the main purpose of generating high spatiotemporal resolution terrestrial LE products. The following conclusions were derived:

(1) The validation results indicated that the MS-PT algorithm provided reliable LE estimations. In particular, the GF-1 LE product with higher resolution showed a better consistency with EC observations compared to the MODIS LE product.

(2) The comparison of three fusion schemes showed that fusion of GF-1 and MODIS NDVI and then computing LE (Scheme 2) can achieve better accuracy and provide more detailed information for retrieving LE variations compared to the fusion of surface reflectance (Scheme 1) and LE products (Scheme 3).

(3) Scheme 2 can produce a time series of LE, which generally corresponds well with MODIS LE and ground measurements, but also has the potential to minimize the biases. The statistical analysis showed that the R^2^ increased from 0.78 to 0.84; the RMSE dropped by 23.4% on average.

Further improvements will focus on the application of the fusion approach over different regions at forested and grassland sites to examine the performance. Other spatiotemporal data fusion models should be conducted to evaluate the utility of LE product fusion in our future study.

## Figures and Tables

**Table 1:** Specification of spectral bands of Chinese GF-1 and MODO9GA products.

Satellite	Sensor	Band Num.	Band Name	Spectral Range (nm)	Spatial Resolution (m)	Revisit Period
GF-1	PMS	1	Panchromatic	450–900	2	4 day
		2	Blue	450–520	8	
		3	Green	520–590		
		4	Red	630–690		
		5	Near Infrared	770–890		
	WFV	1	Blue	450–520	16	
		2	Green	520–590		
		3	Red	630–690		
		4	Near Infrared	770–890		
MODIS	Terra	1	Red	620–670	500	daily
		2	Near Infrared	841–876		
		3	Blue	456–479		
		4	Green	545–565		

**Table 2:** Statistical comparison of the MODIS, GF-1 LE product and predicted LE for Scheme 2.

	Site 1			Site 2		
	R^2^	RMSE	Bias	R^2^	RMSE	Bias
**MODIS LE**	0.83	22.32	11.86	0.78	26.39	10.41
**GF-1 LE**	0.78	16.85	−5.19	0.83	21.98	6.91
**predicted LE**	0.84	15.04	−2.94	0.83	17.42	−0.61

## Appendix A. Details of the ESTARFM Method

In the ESTARFM algorithm, two periods of GF-1/MODIS images (at same or close date) and one additional MODIS image (at the predicted date) are used to calculate the weighting factor $\left(W_{i}\right)$ and the conversion coefficient ($V_{i}$), the final predicted GF-like resolution image can be given by:

(A1) $$G\left(x_{w/2},y_{w/2},t_{p}\right)=G\left(x_{w/2},y_{w/2},t_{k}\right)+\overset{N}{\underset{i=1}{\sum }}W_{i}*V_{i}*\left(M\left(x_{i},y_{i},t_{p}\right)-M\left(x_{j},y_{j},t_{k}\right)\right),$$

(A2) (k=m,n),

where *G* and *M* denote the GF-1 and MODIS pixel value, ($x_{w/2},y_{w/2}$) is the predicted central pixel, $\left(x_{i},y_{i}\right)$ is the location of the *i*th similar pixel, *W* is the size of search window, *N* is the number of similar pixels within the search window.

The temporal weight at $t_{m}$ and $t_{n}$ can be calculate as following:

(A3) $$t_{k}=\frac{1/\left|\sum_{j=1}^{w}\sum_{i=1}^{w}M\left(x_{j},y_{i},t_{k}\right)-\sum_{j=1}^{w}\sum_{i=1}^{w}M\left(x_{j},y_{i},t_{p}\right)\right|}{\sum_{k=m,n}\left(1/\sum_{j=1}^{w}\sum_{i=1}^{w}M\left(x_{j},y_{i},t_{k}\right)-\sum_{j=1}^{w}\sum_{i=1}^{w}M\left(x_{j},y_{i},t_{p}\right)\right)},$$

The weight factor $\left(W_{i}\right)$ is the weight of the *i*th similar pixel, which determined by the geographic ($d_{i}$) and spectral similarity ($R_{i}$) between the *i*th similar and central pixel.

(A4) $$W_{i}=\left(1/D_{i}\right)/\overset{N}{\underset{i=1}{\sum }}\left(1/D_{i}\right),$$

(A5) $$D_{i}=\left(1-R_{i}\right)*d_{i},$$

(A6) $$d_{i}=1+\sqrt{{\left(x_{w/2}-x_{i}\right)}^{2}+{\left(y_{w/2}-y_{i}\right)}^{2}}/\left(W/2\right),$$

(A7) $$R_{i}=\frac{E\left[\left(G_{i}-E\left(G_{i}\right)\right)\left(M_{i}-E\left(M_{i}\right)\right)\right]}{\sqrt{D\left(G_{i}\right)}*\sqrt{D\left(M_{i}\right)}},$$

## Appendix B. Details of the MS-PT Algorithm Logic

The MS-PT algorithm separates the total LE into the unsaturated soil evaporation ($LE_{s}$), the canopy transpiration ($LE_{c}$), the canopy interception evaporation ($LE_{ic}$) and the saturated wet soil surface evaporation ($LE_{ws}$).

(A8) $$LE=LE_{s}+LE_{c}+LE_{ic}+LE_{ws},$$

(A9) $$LE_{s}=\left(1-f_{wet}\right)f_{sm}\alpha \frac{\Delta }{\Delta +\gamma }\left(R_{ns}-G\right),$$

(A10) $$LE_{c}=\left(1-f_{wet}\right)f_{v}f_{T}\alpha \frac{\Delta }{\Delta +\gamma }R_{nv},$$

(A11) $$LE_{ic}=f_{wet}\alpha \frac{\Delta }{\Delta +\gamma }R_{nv},$$

(A12) $$LE_{ws}=f_{wet}\alpha \frac{\Delta }{\Delta +\gamma }(R_{ns}-G),$$

(A13) $$f_{sm}{=ATI}^{k}={\left(\frac{1}{DT}\right)}^{DT/DT_{max}},$$

(A14) $$f_{wet}=f_{sm}{}^{4},$$

(A15) $$f_{c}=\frac{{NDVI-NDVI}_{min}}{{NDVI}_{max}{-NDVI}_{min}},$$

where $f_{sm}$ is soil moisture constraint and can be derived from apparent thermal inertia (ATI), $DT_{max}$ describes the maximum diurnal air temperature range (40 °C), $f_{wet}$ is the relative surface wetness, $f_{T}$ represents plant temperature constraint $\left(\exp(-(T_{a}-T_{opt}\right)/T_{opt}{)}^{2}$), $T_{opt}$ is an optimum temperature (25 °C), $R_{ns}$ is the surface net radiation to the soil $(R_{ns}=R_{n}\left(1-f_{c}\right)$). where G is soil heat flux ($\mu R_{n}\left(1-f_{c}\right)$, μ = 0.18), $R_{nv}$ represents the surface net radiation to the vegetation ($R_{nv}=R_{n}f_{c}$), $f_{v}$ is the vegetation cover fraction and $NDVI_{min}$ and $NDVI_{max}$ are the minimum and maximum *NDVI*, respectively.
